# The Role of Microfinance in China’s Rural Public Health: Evidence from the Anti-Poverty Microcredit Program

**DOI:** 10.3390/ijerph191710872

**Published:** 2022-08-31

**Authors:** Benjian Wu, Yi Cui, Yushuo Jiang

**Affiliations:** 1 School of Economics, Minzu University of China, Beijing 100081, China; 2 School of Economics, Beijing Technology and Business University, Beijing 100048, China

**Keywords:** microcredit, rural public health, loan repayment, poverty, social network

## Abstract

This study presents nonlinear evidence of the effects of a microcredit program implemented in poverty-stricken villages in China on rural public health using multivariate-ordered Probit and IV-ordered Probit models. The results, which were based on a unique set of data gathered from two rounds of official tracking statistics obtained through investigation (2015 and 2018) at a household level, suggest that rural residents’ health levels and health insurance demands are related to the formal credit amount that they receive from the microcredit program. Further, the amount of debt that remains to be paid is a negative mediator and the poverty reduction degree is a positive mediator for the health impact of credit. After dividing the sample into subgroups according to income, credit rating and social network, the results show heterogeneity: the health outcomes of groups with a low income, a high credit rating and a strong social network are more significantly improved by loans. The estimations are still robust after using network and village clan numbers as instrumental variables to address endogeneity. Although most of the existing literature demonstrates that credit and indebtedness have negative impacts on health, our results supplement previous findings of the positive causality between access to formal credit and rural public health by showing that the former can exert positive effects by relaxing individuals’ external constraints and increasing health spending.

## 1. Introduction

Health gaps exist and are widening between the most and least affluent groups in society [1]. The literature on the determinants of these health gaps is extensive, but one general conclusion is that health is determined by social, economic and environmental factors [2]. Research results suggest that wealthier families are better able to protect themselves against the shock of disease or illness [3,4]. This means that initiatives such as microcredit have the potential to improve public health [5]. Indeed, several studies have verified the association between individual health and microcredit, debt and arrears [6,7,8,9]. However, many existing studies have focused on the negative health impact of over-indebtedness, or the reverse effect of health shocks on access to financial markets. Therefore, the causal impact of microfinance on health, and the mechanisms that lead to this impact, need to be empirically explored.

This study aims to explore the potential role of microcredit in improving health-related outcomes. Microcredit has spread rapidly and widely in many countries, aiming to reduce poverty by providing the poor with new opportunities [10] and risk cover tools. Evidence shows that microcredit contributes to poverty reduction [11], and it also aims to increase the access of poor communities to health care [12] and education, and enhance social capital [13,14]. Due to microcredit, the income level of recipients increases, raising their private medical expenditures and health inputs [15], and result in improved health outcomes [16,17]. On the contrary, a lack of financial resources may lead to unhealthy coping mechanisms and individuals may cut back on the costs of healthcare and medicines [9,18].

A recent study shows that microcredit and health are interrelated in diverse ways [19], through different mechanisms [19,20,21,22]. Although microcredit can theoretically promote health, taking on debt may also adversely affect health [18,23]. The onset of indebtedness is related to the deterioration of mental [24,25,26,27] and physical health [28,29,30], which in turn might worsen overall welfare [9,31,32]. These negative effects have been attributed to financial strain [29]. Alternatively, debt accumulation minimizes the accessibility of forthcoming resources for health-related investments [25,33,34]. The effects are also influenced by other factors such as the source of debt [35], the interest rate attached to the debt [36] and the repayment structure.

Furthermore, the causal pathway detected in existing studies varies [37]. The causation between debt and individual health is likely to run in both directions [35]: some measures of microcredit are found to affect health, whereas other measures of health are found to affect microcredit [27]. The former is derived from the economic causation hypothesis, which assumes that microcredit affects health [38]. The latter is associated with the health selection hypothesis, which states that health affects microcredit. Some additional variables, such as major life events, family background or genetic endowment, may lead to a deterioration of health and microcredit access [39,40]. So far, it is unclear which causal mechanisms (such as social causation, debt amount or family background factors) are relatively more important [41]. This has made designing interventions difficult, leaving causality unresolved.

Although several studies have shown an association between loans (debt) and individual health [7,8], the determination of a causal relationship is often hampered by methodological problems. First, the vast majority of studies are based on cross-sectional data [42] and thus are unable to monitor the dynamic effects, while studies using longitudinal data are generally less likely to utilize standardized health measures [27]. Second, many studies rely on survey data, self-rated measures of health and problematic subjective measures of debt, which are prone to measurement bias [23,42]. Third, most studies do not deal with the problem of two-way causality [42]. Thus, more longitudinal research is needed to establish the potential mechanisms and mediators of this relationship and to determine the causal relationship.

Given the above background, it is clear that there is a vast body of literature available on the association between external debt and health. However, none of the studies have explored the simultaneous effects of microfinance and debt on health outcomes. Different social, cultural and political contexts also affect the microfinance–health connections. This study aims to explore the consequences of microcredit and indebtedness on rural public health outcomes in China.

## 2. Institutional Background

In China, approximately 55 million rural residents were in poverty at the end of 2015. Illness is one of the main causes of poverty among the rural population: poverty caused by illness and poor health accounted for 44.1% of the total poverty-stricken population (i.e., more than 20 million people), including 7.34 million people suffering from serious and chronic diseases (National Health Commission of China, 2015). Due to the scarcity of funds, medical expenditure of the poor is limited. Under this circumstance, microcredit is a common tool adopted by the government to reduce the vulnerability to poverty. Through providing funds to poor households, the microcredit program can relax the financial constraints and improve the health-care expenditure of rural residents, and it is widely used in poverty reduction in various countries.

Financial services such as credit are rare in rural China. On the one hand, commercial finance institutions tend to raise the threshold and interest of formal credit to the poor considering the insolvency risk of the debtors, or they reduce credit supplies under the same conditions; on the other hand, the small-scale peasant economy has its common methods of risk diversification, such as diversified planting or informal credit. When facing excessive credit interest rates or strict credit ratings, farmers are unlikely to participate in credit programs. This has been regarded as the “Insufficient Supply and Demand” phenomenon in the rural finance market.

In view of the above facts, a market-oriented loan supply would inevitably lead to credit rationing and insufficient credit availability for the poor. Therefore, financial development needs government support. In the past decades, the Chinese government has implemented a series of actions to promote financial development and improve residents’ welfare in rural areas, such as providing subsidized loans to the poor and creating new agricultural insurance products. In 2012, the State Council Leading Group Office of Poverty Alleviation and Development of China identified 14 extreme poverty regions to achieve the task of poverty eradication, including 680 poverty-stricken counties. In 2011, the per capita net income of rural residents in these extreme poverty regions was 4191 yuan, only 60.1% of the national rural average level. The poverty rate in these regions is 28.4%, 15.7 percentage points higher than the national average. The natural conditions in these areas are poor, and the poverty-causing factors are complex. To help poverty-stricken households get rid of poverty, the Chinese government established a series of targeted supportive policies related to microfinance. Among poverty-stricken villages, Yanchi County in Ningxia Hui Autonomous Region is an example in which the problem of poor households’ loan difficulty has been solved. Yanchi is a county located in Northwest China with about 160 thousand permanent populations, including 139 thousand agricultural populations. In 2014, to alleviate poverty, Yanchi County promoted the microcredit program of providing credit without guarantee or mortgage for the poverty-stricken population. In 2015, the microcredit program was fully implemented. By increasing loan interest subsidies, improving credit cultivation mechanisms and broadening channels of financial intermediation, Yanchi County attempted to solve the problems in rural finance and achieved great progress in microfinance innovation. Low-income households were encouraged to obtain microcredit of less than 50 thousand yuan within three years. For the officially registered poverty-stricken households, the interest rate of microcredit is fully subsidized by the government. The fiscal and financial efforts of Yanchi County government have brought great achievements in poverty reduction. In 2015, more than 61 thousand people have been lifted out of poverty. By 2016, the balance of poverty alleviation microcredit loans in Yanchi County had reached 3.14 billion yuan, covering more than 28,000 loan households. At the end of 2017, the total amount of poverty alleviation microcredit funds in Yanchi County increased from 2.8 billion yuan to 3.5 billion yuan, and the number of beneficiaries increased from 20 thousand to about 30 thousand. Before the implementation of the microcredit program in 2015, the poverty rate of Yanchi County is more than 20%, much higher than the provincial level of 12.5% and the national level of 8.5%. However, in 2018, after three years of the microcredit program pilot, the poverty rate of Yanchi County decreased to 3.2%, almost equaling the national average of 3.1% in the same period. The microcredit program breaks down the barriers that prevent vulnerable people from accessing financial services, and it has achieved remarkable outcomes, effectively addressing the shortage of funds among poor families.

However, despite the remarkable achievements in poverty reduction, Yanchi County is still a poor, vulnerable region: 74 of the 102 administrative villages remain in poverty. Among the 139 thousand agricultural population, there are 11,228 officially registered poverty-stricken households with a total of 34,046 people. Even among the people who have been lifted out of poverty, the phenomenon of returning to poverty due to illness and accidents is serious.

We choose Yanchi County as our sample area for the following two reasons. First, Yanchi County is one of the 680 poverty-stricken counties identified by the Chinese government and thus, many poverty alleviation programs, such as microfinance, have been piloted in this region. In addition to the microcredit programs, rural residents in Yanchi County have almost no access to credit. Therefore, the credit amount information collected from the official tracking statistics in rural China is approximately equal to the total credit amount of the poor. Second, the poverty rate caused by residents’ health problems in Yanchi County is extremely high, amounting to nearly 40% of the poverty-stricken population. Therefore, the sample of Yanchi County is representative of the poor in the study of the microcredit–health causal relationship in rural China. By increasing loan interest subsidies, improving credit cultivation mechanisms and broadening channels of financial intermediation, Yanchi County attempted to solve the problems in rural finance and has achieved great progress in microfinance innovation.

There are many theoretical and practical studies on the rural public health improvement performance of microfinance. Research shows that the increase in debt has a negative impact on the economy [43], which in turn leads to a decline in health outcomes [44]. Financial strain might limit people’s access to health services, while predatory lending practices targeted at low-income areas could also exacerbate health problems [45]. There was also found to be an increased impact of high-interest debt on health deterioration, which was much more substantial and more significant for lower-income groups [36]. The introduction of microfinance in Southeast Asian countries in 2013 has broken down the barriers that prevent vulnerable people from accessing financial services. Therefore, microfinance is an important influencer of health.

However, microcredit and related programs have also become the subject of increasing criticism. In fact, some even claim that the microfinance industry is currently facing an ethical crisis [46]. Although it brings low-income groups more opportunities to seek medical treatment, microcredit may also intensify health problems among the poor. For instance, the factors include a high-interest debt repayment structure, difficulties in debt repayment [47], worry-exacerbating debt problems and financial concern [48].

Some studies have linked social networks [49], social capital [50] and social pressure [51] with microfinance. The social environment significantly affects the availability of credit. Due to a lack of credit support, financial stress was found to be higher among families from ethnic minorities [52]. Nevertheless, there has been little research on the relationship between the gap in the availability of social networks and microfinance due to the difficulty of quantifying social capital. Nahapiet and Ghoshal [53] divided social capital into three categories: structural social capital, relational social capital and cognitive social capital. Recently, some studies have proxied social networks through measurements such as generalized social trust or various cultural dimensions [54,55,56], and further predict the effects of social networks on microcredit performance. However, seldom have studies looked at how rural public health is determined by microcredit availability and the role of social networks in the above causal chain. Building on other scholars’ research [53], we argue that the microcredit program in rural areas is facilitated by social determinants such as local clanship or kinship networks (which are relational social capital) and hypothesizes that microcredit availability helps improve rural public health by relaxing the health expenditure constraints of rural residents.

The choice of health outcome measurements is another debate in the existing literature. Previous research shows that the measurement of health variables is important in analyzing the impact of microcredit on health. The results can vary greatly depending on how health is measured [57]. Most previous studies used two common proxies: premature mortality and life expectancy rates at birth [6,37,58]. However, these sample-level data cannot accurately measure the health level of the individual. In this study, we use microcredit and health data at the individual level to evaluate the health impact of microcredit programs, as well as to detect the mechanisms.

## 3. Data and Methodology

### 3.1. Data

Evidence on the long-term rural public health improvement impacts of the microcredit program for poverty-stricken people is scarce. We used a unique 3-round panel (2015, 2018 and 2019) dataset on rural households of Yanchi, a poverty-stricken county in Northwest China, that includes information about microcredit, social networks, health level, health expenditure, household characteristics and family income. The data for 2019 did not include comprehensive information on individual or household characteristics; thus, we did not include this round of data in the analysis as a sample year. We only used some of the variables for the year 2019, such as the self-reported health level and health insurance purchased by the individuals, to overcome the possible two-way causal effects between microcredit and individual health. The sample areas in this study were purposively selected from the most poverty-stricken areas in Northwest China, and they represent the credit availability and health situations of the poverty-stricken households in rural China well. We selected more than 100 villages with large-scale poor households in the sample county, then investigated all the officially registered poverty-stricken households. The questionnaire includes basic information of the households and the farmers, agricultural production, health-related information, credit rating, village social norms and government supports (such as subsidies and agricultural training).

In 2014, Yanchi County accurately identified 11,203 officially registered poverty-stricken households and carried out poverty alleviation actions such as fiscal and financial poverty alleviation programs. The financial poverty alleviation programs of Yanchi County were carried out early in 2015, and it was one of the first pilot regions in rural China to implement microfinance programs. By providing microcredit to poor households, the programs improved the credit availability of rural residents and reduced their financing costs. The administrative data of this study included information covering all 11,228 poverty-stricken households (increased by 25 households from 2015 after an accurate identification recheck) in 109 villages in 9 towns of Yanchi County.

The base period data of poverty households were collected in 2015. In 2018, we conducted a follow-up survey in Yanchi County and obtained the tracking data of the samples. In order to further understand the dynamic changes among poor households and better analyze the performance of rural public health improvement, we obtained information on the health level, health insurance expenditure, family income, debt amount and poverty status of 11,228 poor households in 2019 through another round of the small-scale survey. The survey collected detailed information on a number of socio-economic variables, including the household demographics, income structure, health level, credit and insurance and the basic situation of the household population, in 2015, 2018 and 2019. Because the officially registered poverty-stricken households were precisely tracked by the county government, the attrition rate of our sample was zero, providing us with a set of balanced panel data. The individual-level panel data with the two phases of the 11,228 households included information on the production and life of the poor households mentioned above, and they could thus comprehensively reflect the living conditions and poverty status of poor households. We use the data to measure the dynamic change in health level and health insurance purchase behavior of rural residents after participating in the microcredit program. Descriptive statistics of the sample regarding the heads of rural households in Yanchi County are presented in Table 1.

The first measure that we consider for rural residents’ health is the health level of the household head. We use self-rated health to measure the health level of household heads: respondents were asked about self-assessed health levels and health insurance purchased by all members of the household. The scoring standard is as follows: 4 = in good health and seldom gets sick; 3 = in average physical condition and sometimes gets sick; 2 = with a history of chronic diseases; 1 = in a disabling condition.

However, while simple to understand, the self-rated health level is not particularly informative because of its categorical nature. Besides, an individual’s self-reported health status is subjectively affected by their social and cultural background, given the individual’s subjective health. To obtain more objective health-related measurements, we also asked the respondents additional questions about their health status: the amount of health insurance and elder insurance purchased by a household. The premium and coverage of life insurance were also collected to measure the expenditure of rural residents on health-related insurance. These variables are likely to contain more information on an individual’s attention to health. The outcome variables contained information on individual health in 2019.

The two measures of credit availability were whether the household obtained formal credit in 2015/2018 and the amount of loan obtained by the household in 2015/2018. Because the credit availability of farmers is endogenous, we introduced the social network of the individual and the cultural background of the village as variables. An empirical challenge in our context is characterizing the social network of each household. The measure of a strong/weak network can be interpreted as whether the randomly selected families are from the main clans of the village. Another measure, the number of clans in the village, could account for the differences in relative size or strength among clans.

The key to our analysis was determining the powerful clans in each village. Importantly, we had access to the non-anonymized version of the administrative dataset and had full names (the first and family name) for every individual in the sample. We can measure the large-scale family networks in Yanchi County due to naming conventions with two characteristics: (a) within a village, a shared family name implies family relations, and the first names of close relatives of the same generation are usually similar (for example, a certain word in the first name is the same); (b) many villages are named after the most popular surname name among the villagers. Given the full names of all individuals in a village, we can also reconstruct all of the edges in the lineage network by examining the joint occurrences of family names and the same parts of the first names; we could also obtain the clan network by calculating the household surname population size in the village. As a result, we are able to observe ties between families merely by the occurrence of the surname and first names within an individual.

The question that we consider in this paper is whether access to microcredit has a positive effect on the health outcomes of rural residents. We hypothesize that persons who participate in the microcredit program will have a higher health level and/or investment intention regarding health insurance. Our two-round micro survey data allow us to overcome many of the above methodological problems. First, we overcome the limitations of cross-sectional data by using an intertemporal dataset, which allows us to measure the effects of previous microfinance program participation (2015/2018) on subsequent health-related outcomes (2019). We obtained the year in which participants obtained formal credit aid as well as the credit amount in each year from the Poverty Alleviation Office. We also had data available for the full poverty-stricken population in Yanchi County, which allowed us to compare the health status of individuals who obtained loans from microfinance programs to the health status of individuals who had not yet participated in the program.

Second, we use countywide administrative individual-level data at the individual level on the demand for health insurance, microfinance and financial assistance. These data also enable us to use standardized measures of health levels and observe these measures for the entire poverty-stricken population in the sample county.

Third, we obtained an objective measure of microfinance within the sample range: we focus on the microfinance program implemented in Yanchi County. The program provides low-interest loans to the poor, while the decision of whether to participate in the program and apply for the loans is made by the individual. We use information about whether rural residents obtain loans from the program and the number of loans that they receive to measure their access to microfinance. Because financial institutions are reluctant to offer loans to poor households due to risk aversion, the microfinance program provides a clear measure of loan access and loan amount. Hence, the institutional context that we study facilitates identification without the significant risk of bias as in most other studies of the loan–health relationship.

Lastly, we also used detailed administrative data on various other relevant variables. Although the measurement of the relationship between microfinance and health may be affected by endogeneity issues, detailed information on a household and individual level regarding, for example, negative life events (e.g., poverty, disability, illness), other socio-economic variables (e.g., income, social network, village clans), household characteristics (e.g., credit rating (The credit rating standard in Yanchi County is based on the proportion of 10% spiritual civilization, 50% credit situation, 30% family assets and 10% basic situation. The loan limit and interest rate are all correlated with credit rating), insurance participation, household structure) and personal characteristics of household heads (e.g., gender, age, education, skills, off-farm working) are conducive to some selection bias elimination that has hindered earlier research. We use these variables to control for important differences in household and individual characteristics that may influence the microfinance–health relationship.

### 3.2. Estimation Methodology

#### 3.2.1. Benchmark Model

The explanatory variables of this paper are rural residents’ self-rated health level in 2019 ($Health_{ihv}$) and health insurance purchased in 2019 ($Health\_ins_{ihv}$). $Health_{ihv}$ is an ordered discrete variable with values ranging from 1 to 4, and $Health\_ins_{ihv}$ is an ordered discrete variable with values ranging from 0 to 9. Therefore, in order to explore the impact of microcredit (credit availability and loan amount) on rural residents’ health levels and health insurance participation, this paper constructs the following ordered Probit measurement models as a benchmark:

(1) $$Health_{hv}=\alpha_{0}+\alpha_{1}Loan\_dum_{hv}+\alpha_{2}X_{ivt}^{\prime }+\alpha_{3}X_{hvt}^{\prime }+\alpha_{4}X_{vt}^{\prime }+\eta_{v}+\varphi_{t}+\varepsilon_{ivt}$$

(2) $$Health_{hv}=\alpha_{0}+\alpha_{1}Ln{\left(loan\right)}_{hv}+\alpha_{2}X_{ivt}^{\prime }+\alpha_{3}X_{hvt}^{\prime }+\alpha_{4}X_{vt}^{\prime }+\eta_{v}+\varphi_{t}+\varepsilon_{ivt}$$

where $Health_{hv}$ is the self-rated health level of the head of household h in 2019. $Health_{hv}$ is an ordered discrete variable with values of 1 to 4. $Loan\_dum_{hv}$ denotes whether or not household h obtained microcredit in 2015. $Ln{\left(loan\right)}_{hv}$ is the logarithm of the income of household h in village v. $X_{ivt}^{\prime }$ corresponds to a full set of individual covariates such as age and education, $X_{hvt}^{\prime }$ are household-level factors such as household demographics and wealth, $X_{vt}^{\prime }$ are village-level factors such as village clanship, $\eta_{v}$ are the village fixed effects, $\varphi_{t}$ are year fixed effects and $\varepsilon_{ivt}$ are the error terms. The fixed effects of region and timing are used to reduce potential selection biases due to time-invariant unobserved heterogeneity and individual trends therein. All monetary variables are deflated by the producer price index.

(3) $$Health\_ins_{hv}=\beta_{0}+\beta_{1}Loan\_dum_{hv}+\beta_{2}X_{ivt}^{\prime }+\beta_{3}X_{hvt}^{\prime }+\beta_{4}X_{vt}^{\prime }+\eta_{v}+\varphi_{t}+\varepsilon_{ivt}$$

(4) $$Health\_ins_{hv}=\beta_{0}+\beta_{1}Ln{\left(loan\right)}_{hv}+\beta_{2}X_{ivt}^{\prime }+\beta_{3}X_{hvt}^{\prime }+\beta_{4}X_{vt}^{\prime }+\eta_{v}+\varphi_{t}+\varepsilon_{ivt}$$

where $Health\_ins_{hv}$ is the amount of health insurance purchased by household h in 2019. $Health\_ins_{hv}$ is an ordered discrete variable with values of 0 to 9. The definitions of other variables are the same as above. Equations (1)–(4) are estimated separately for both dependent variables. Standard errors are clustered at the village level.

#### 3.2.2. Exogenous Event

To claim a causal relationship between microcredit and health, the event studied must be exogenous. However, access to microcredit may not be fully exogenous in general. In fact, studies have shown that the causal relationship between microcredit and rural public health may be inherently reversed [8]. In addition to reverse causal bias, unobservable attributes may also affect microcredit and personal health. We introduce social network indicators to overcome the endogeneity problems.

(5) $$Loan\_dum_{hv}=\theta_{0}+\theta_{1}Clanship_{v}+\theta_{2}Network_{hv}+\theta_{3}X_{hvt}^{\prime }+\theta_{4}X_{vt}^{\prime }+\eta_{v}+\varphi_{t}+\varepsilon_{ivt}$$

(6) $$Health\_ins_{hv}=\gamma_{0}+\gamma_{1}Loan\_dum_{hv}+\gamma_{2}Z\prime +\gamma_{3}X_{ivt}^{\prime }+\gamma_{4}X_{hvt}^{\prime }+\gamma_{6}X_{vt}^{\prime }+\eta_{v}+\varphi_{t}+\varepsilon_{ivt}$$

$Network_{hv}$ is measured as kinship and clanship networks, as described in Table 1. $Clanship_{v}$ is the clan number in the village, which denotes the clanship at the village level. The basic regression controls for basic demographic characteristics (such as household structures, gender, age and health status) and all available measures for on-farm and off-farm activities.

In the first step, we regress the dependent variables on the instruments, which is shown in Equation (5). Then, in the second step, in testing a particular theory, we learn from the existing literature [59] and re-estimate Equation (1) by adding a set of exogenous explanatory variables, Z′, which is shown in Equation (6).

We also introduce other dependent variables related to rural public health for robustness checks. First, the amount of elder insurance purchased by the household $Elder\_ins_{hv}$. Second, the insurance coverage of health insurance purchased by the household $Ln{\left(coverage\right)}_{hv}$. Third, the insurance premium of health insurance purchased by the household $Ln{\left(premium\right)}_{hv}$. The estimation models are as follows:

(7) $$Elder\_ins_{hv}=\gamma_{0}+\gamma_{1}Loan\_dum_{hv}+\gamma_{2}Z\prime +\gamma_{3}X_{ivt}^{\prime }+\gamma_{4}X_{hvt}^{\prime }+\gamma_{6}X_{vt}^{\prime }+\eta_{v}+\varphi_{t}+\varepsilon_{ivt}$$

(8) $$Ln{\left(coverage\right)}_{hv}=\gamma_{0}+\gamma_{1}Loan\_dum_{hv}+\gamma_{2}Z\prime +\gamma_{3}X_{ivt}^{\prime }+\gamma_{4}X_{hvt}^{\prime }+\gamma_{6}X_{vt}^{\prime }+\eta_{v}+\varphi_{t}+\varepsilon_{ivt}$$

(9) $$Ln{\left(premium\right)}_{hv}=\gamma_{0}+\gamma_{1}Loan\_dum_{hv}+\gamma_{2}Z\prime +\gamma_{3}X_{ivt}^{\prime }+\gamma_{4}X_{hvt}^{\prime }+\gamma_{6}X_{vt}^{\prime }+\eta_{v}+\varphi_{t}+\varepsilon_{ivt}$$

#### 3.2.3. Mediation Model

To document the mediating mechanisms of the impact of microcredit on rural public health, we re-estimate Equation (1) by adding a set of mediating explanatory variables $M_{hv}$:

(10) $$Health_{hv}=\alpha_{0}+\alpha_{1}Loan\_dum_{hv}+\alpha_{2}M_{hv}+\alpha_{3}X_{ivt}^{\prime }+\alpha_{4}X_{hvt}^{\prime }+\alpha_{5}X_{vt}^{\prime }+\eta_{v}+\varphi_{t}+\varepsilon_{ivt}$$

where $M_{hv}$ is the mediator variable derived from theory, through which debt and poverty status would affect the health outcomes of poor households. In this paper, we consider the amount of debt of household h in 2019 ($Ln{\left(debt\right)}_{hv}$) and the poverty relief status of household h in 2019 ($Poverty\_relif_{hv}$) as mediator variables.

Finally, the estimated microcredit demand model of individual i in village v at year t is shown in Equation (8):

(11) $$M_{hv}=\sigma_{0}+\sigma_{1}Health_{hv}+\sigma_{2}X_{v}+\eta_{v}+\varphi_{t}+\varepsilon_{ivt}$$

where $M_{hv}$ are the mediators and, in this paper, we measure this variable with debt amount ($Ln{\left(debt\right)}_{hv}$) and poverty relief ($Poverty\_relif_{hv}$). Similar to Equations (1) and (2), two measures of health are used: (a) health level reported by the individual and (b) health insurance purchased by the household.

## 4. Results

Focusing on a microfinance program implemented in rural counties, we find that persons who participate in the microfinance program and receive credit support have higher health levels and/or investment intention for health insurance. We also find that both health level and health insurance demand increase significantly for individuals participating in rural microfinance programs. Furthermore, the individual health level of “in good health and seldom gets sick” ($Health_{hv}$ = 4) increased by 7.2% and the probability of rural residents not purchasing health insurance ($Health\_ins_{hv}$ = 0) decrease by 6.3% after acquiring debt. Overall, we find that obtaining microcredit helps to improve health, yet debt is associated with ill health. This section will expand on these results.

### 4.1. Benchmark

This paper uses the ordered Probit model to estimate the impact of microcredit on rural residents’ health under the control of education level, family income, household structure and other related factors. Columns 1~4 of Table 2 report the maximum likelihood estimation results of the impact of microcredit on rural residents’ health. The coefficients of both microcredit availability ($Loan\_dum_{hv}$) and loan amount ($Ln{\left(loan\right)}_{hv}$) are positive at the significance level of 1%, which shows that, under the control of education level, family income, poverty situation and other factors, microcredit participation has a significant impact on rural residents’ health. Specifically, microcredit availability and subsidized loans would increase the self-rated health level and health insurance participation of rural residents.

Given that the parameter definition of the ordered Probit model is not intuitive, the above estimation results can only provide limited information on the direction and significance of the health impact of microcredit, and they cannot determine the specific numerical value of the impact. Therefore, to compare and analyze the probability of the impact of microcredit on rural residents’ health, we further calculated the marginal effect of each value. The results are reported in Table 3.

The marginal impact of microcredit on the rural residents’ self-rated health level, denoted as “in a disabling condition” ($Health_{hv}$ = 1), “with a history of chronic diseases” ($Health_{hv}$ = 2) and “in average physical condition and sometimes gets sick” ($Health_{hv}$ = 3) is negative at the significance level of 1%. The health level of “in good health and seldom gets sick” ($Health_{hv}$ = 4) is positive at the significance level of 1%. Specifically, compared with the residents who did not participate in the microcredit program, the probability of rural residents who obtained microcredit and reported a health level of 1, 2 and 3 decreased by 2.3%, 0.9% and 4.1%, respectively. However, the marginal impact of microcredit on rural residents with a reported health level of 4 is significantly positive at the significance level of 1%. This indicates that, compared with the residents who did not participate in the microcredit program, the probability of rural residents who obtained microcredit and reported a health level of “in good health and seldom gets sick” increased by 7.2%. We can thus conclude that participation in microcredit would decrease the probability of a low health level and increase the probability of a high health level.

Similarly, after microcredit participation, the probability of rural residents not purchasing health insurance ($Health\_ins_{hv}$ = 0) decreased by 6.3%, while the probability of purchasing other amounts of health insurance ($Health\_ins_{hv}$ = 1~9) increased more or less. Therefore, microcredit also improves rural residents’ health insurance participation.

### 4.2. Addressing Endogeneity

There are different strategies utilized to address endogenous problems in the literature related to credit and finance availability. In rural China, an important feature of Chinese clan culture is the inheritance and continuation of kinship, which is embodied as a type of cultural norm. Because the individual surname representing clan networks at the village level is not affected by economic factors, in some studies, this was considered as an exogenous factor of economic decision-making. For example, Peng (2004) took the proportion of people with the same surname in a village as the explanatory variable to study the role of the social network in the development of rural economics [60]. Some other scholars attempted to address endogeneity by taking “the regional difference of the historical clan network” as an instrumental variable to check the benchmark robustness.

Here, we take both village-level clanship indicators and household-level network indicators as instruments to evaluate the microcredit availability and loan amounts of rural residents. Specifically, we take the “number of main surnames in village v” ($Clanship_{v}$) as the village-level clanship index to denote the multiplicative inverse of social fractionalization. When most of the people in a village belong to a clan according to their surname, and the number of main surnames is small, then the concentration of the village social network is high. When the surnames of a village are scattered and the proportion of people with the main surnames is relatively low, then the concentration of the village social network is low. This is strongly correlated with households’ access to microcredit or loan amounts in the village, but less strongly correlated with rural residents’ health level or health insurance purchase behavior. We also take “whether the surname of household h belongs to the main surnames of village v” to measure the household network. This could be regarded as the household-level network index because there is a distinct clan network in rural China, which is usually composed of several main surnames. If the household surname is one of the main surnames in the village, then we consider that the household has a strong network in the village. When the population proportion of this surname is large, the probability that the household has a strong kinship network is even higher; however, if a household does not share the main surnames, the probability that this household has a strong kinship network will decrease as the population with the main surnames increases. Similar to the clan number indicator, the measures of whether the surname of a household belongs to the main surnames at the village level do not seem to affect the health-related outcomes of households. Therefore, these two exogenous instruments can address the endogenous problems caused by selection bias and simultaneity.

Table 4 reports the benchmark results of this paper. We include the two measurement variables of microcredit demand in Equation (2), $Loan\_dum_{hv}$ and $Ln{\left(loan\right)}_{hv}$, respectively. $Ln{\left(loan\right)}_{hv}$ represents the number of loans that a household receives from the formal microcredit program. It is a continuous random variable because we excluded the observations if $Loan_{hv}$ equals zero, i.e., when farmers did not obtain any loan from the microcredit program. $Loan\_dum_{hv}$ is a dummy variable that denotes whether or not a household receives microcredit. Specifically, it equals 1 if a household obtains formal microcredit and 0 if not. We use two-stage CMP ordered Probit estimation to address the endogeneity problem.

Table 4 reports the two-stage estimates of the impact of social networks on households’ access to microcredit and the loan amount, as well as the effect of microcredit on rural public health. From the first-stage estimation (as shown in Equation (5)), the reciprocal of social fractionalization strongly decreases household microcredit availability and loan amount, while the household social network significantly increases microcredit participation. In the second stage, the regression results show that after the introduction of village-level and household-level instruments of social networks, the microcredit participation of households still has a significant positive impact on rural residents’ health. Therefore, the benchmark conclusions of Table 2 are robust.

We then introduce a series of tests to verify the validation of instrumental estimation. The results of the tests are reported in Table 5. The statistic of the weak instrumental variable test (Cragg–Donald Wald F statistic) is approximately 32, greater than the critical value of 19.93 under 10% maximal IV size, thus rejecting the null hypothesis of weak instrumental variables. Further, the unrecognizable test shows that the *p*-value of the Kleibergen–Paap rk Wald F-statistic is 0.0000, strongly rejecting the unidentifiable null hypothesis.

### 4.3. Heterogeneous Analysis

Under the same microcredit availability, the health effects of different individual groups may be heterogeneous. First, we divided the individuals into two groups according to their credit score rating. The range of the credit score in the sample is 1 to 4. As the value increases, rural residents’ credit scores gradually increase. Panel A of Table 6 reports the 2SLS estimation results of the influence of microcredit on the individuals’ health within different credit score groups. We only report the second-stage estimates. According to panel A of Table 6, microcredit has a significantly positive effect on both health level and health insurance demand in the group with high credit scores ($Credit\_score_{hv}=4$). For the group with low credit scores ($Credit\_score_{hv}=1,\;2\;\mathrm{or}\;3$), however, microcredit only plays a role in encouraging rural residents to purchase health insurance, and it has little impact on their health rating level.

Second, we divided the individuals into two groups according to household income to study the impact of microcredit on rural residents with different income levels. In the sample of this study, only 34% of the households had a monthly income of more than 5000 yuan; in other words, households with a monthly income of more than 5000 yuan are relatively affluent groups within the village. Therefore, we took 5000 yuan as the critical value for distinguishing between high-income and low-income groups. By dividing the samples into different income groups, we study the effects of microcredit on the heterogeneity of rural residents’ health outcomes. Generally, high-income groups have a high ability to resist risks, and they are usually responsible for helping low-income members out of poverty. From the perspective of risk sharing, microcredit may increase the economic burden of high-income members. Panel B of Table 6 reports the second-stage estimates of the 2SLS regression of the influence of microcredit on individuals’ health within different income groups. For both groups, microcredit has a positive impact on rural public health. For low-income households, the social network has an even stronger impact on both health levels and health insurance demand. For the high-income group, microcredit has a significant positive effect on residents’ health levels, yet it has no significant effect on health insurance demand. Thus, microcredit has heterogeneous impacts on the health outcomes of different income groups. Specifically, compared with high-income households, the impact of microcredit on low-income households is larger, which means that microcredit in rural China can improve the health status of poor households.

Third, we divided the sample into two groups according to social networks. If the surname of the household head was the main surname in the village, then we denoted the household as a strong network household. If the surname of the household head was not one of the main surnames in the village, we included it in the weak network group. According to panel C of Table 6, microcredit improves health outcomes in rural China mainly by increasing the health levels of individuals in groups with a strong social network and increasing the health insurance demand for individuals in weak social networks.

To compare and analyze the probability of the impact of microcredit on rural residents’ health in different groups, we calculated the marginal effect of each value. The results are reported in Table 7. First, for the high credit score group, the marginal impact of microcredit on the rural residents’ self-rated health level, denoted as “in a disabling condition” ($Health_{hv}$ = 1), “with a history of chronic diseases” ($Health_{hv}$ = 2) and “in average physical condition and sometimes gets sick” ($Health_{hv}$ = 3), was negative at the significance level of 1%. The health level of “in good health and seldom gets sick” ($Health_{hv}$ = 4) was positive at the significance level of 1%. Specifically, compared with the residents who did not participate in the microcredit program, the probability of rural residents who obtained microcredit reporting a health level of 1, 2 and 3 decreased by 2.7%, 1.1% and 5.8%, respectively. However, the marginal impact of microcredit on rural residents’ reported health level of 4 was significantly positive at the significance level of 1%. This indicated that, compared with the residents who did not participate in the microcredit program, the probability of rural residents who obtained microcredit reporting a health level of “in good health and seldom gets sick” increased by 9.6%. Moreover, microcredit had a positive effect by encouraging groups with both high credit scores and low credit scores to purchase more health insurance products.

Second, for both the high-income group and low-income group, microcredit improved residents’ self-rated health levels. Specifically, the probability of indicating a health level of “in good health and seldom gets sick” ($Health_{hv}$ = 4) increased by 5.3% for the high-income group and 10.6% for the low-income group.

Third, after microcredit participation, the probability of rural residents in the weak network group not purchasing health insurance ($Health\_ins_{hv}$ = 0) decreased by 3.1%, while the probability of purchasing health insurance ($Health\_ins_{hv}$ = 1~9) increased. Microcredit improved health insurance participation for individuals in the weak network group. For the individuals in the strong network group, microcredit improved the probability of high health leave.

### 4.4. Mediation Effects

**Figure 1:**
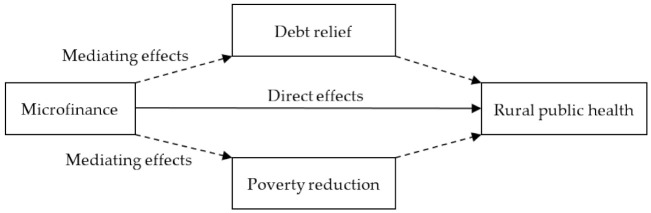
Mediation diagram.

Generally, household debt does not necessarily lead to financial problems. However, if a household is not able to manage its debts, it may fall into indebtedness and financial problems may emerge. We captured the net debt amount and debt service burden of households based on an objective model according to a previous study [61]. Moreover, the poverty status would also affect the utility of microcredit by rural residents, thus acting as another mediator. In this study, we detect the mediating effects of both debt and poverty status in the microcredit–health relationship (see Figure 1).

The KHB method proposed by Breen et al. [62] can be used not only for the mediating effect analysis of a nonlinear probability model, but also for the case of multi-dimensional mediators. In this section, we use the KHB method to estimate the mediation effects of the microcredit–health relationship. Specifically, we test whether the debt or poverty reduction is the channel for microfinance to affect the rural public health.

In this section, we use the KHB method to estimate the mediation effects of the microcredit–health relationship. Through the calculation of the total effect, direct effect and indirect effect by ordered Probit method, we effectively estimate the mediators of the nonlinear regression model. Theoretically, debt and poverty strongly affect rural public health. We developed a poverty indicator, namely whether the household had eliminated poverty in 2019, and a debt indicator, namely the logarithm of household debt in 2019. Here, we estimate the mediation effects of the debt amount in 2019, as well as the poverty relief situation in 2019. The results in Table 8 show the estimates of the reduced model and full model and the difference between the first two estimates. We consider the estimation effect of the reduced model as the total effect, the full model as the direct effect and the difference (‘Diff’) model as the indirect effect. We find that obtaining formal credit could improve the rural health level by 6.21%. Under the control of debt in 2019, the impact of formal credit increased to 7.28%, leaving an indirect effect of −1.06%. The total effect was 1.6 times the direct effect, and 38% of the total effect arose from the amount of liabilities.

We find that the debt amount acts as a negative mediator in the causal relationship between microcredit and health. Although microcredit helps to improve the health level, heavy debt in 2019 had a negative effect on rural public health. Poverty relief status is a positive mediator. If the household escaped poverty, microcredit would have an even stronger effect on rural residents’ health levels, as well as health insurance demand.

### 4.5. Robustness Checks

**Figure 2:**
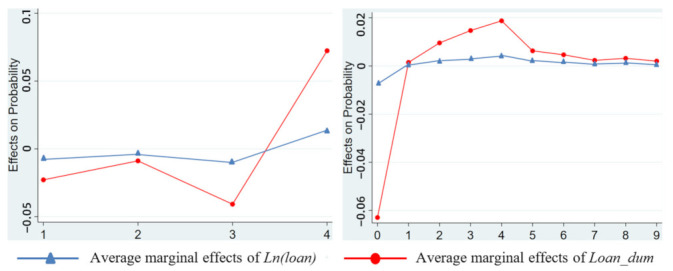
Average marginal effects of microfinance variables and trends with health level (**left**) or health insurance demand (**right**).

For the health level and health insurance demand of rural residents as studied in this paper, the marginal effect is the influence of the variable on the probability of individuals choosing a certain score regarding their health level. “Self-rated health level”($Health_{hv}$) and “health insurance purchase amount” ($Health\_ins_{hv}$) are ordered discrete variables; the former is 1~4, and the latter is 0~9. For the microcredit availability corresponding to each score, each variable will have different marginal effect values. In order to more intuitively analyze the marginal effect of each variable and its change with the values of health outcomes, we visualized it as a graph, as shown in Figure 2.

In the practice of the microcredit program implementation in Yanchi County, the upper age limit for borrowers was 65 before the year 2017. This restriction may affect the causality analysis between microfinance and rural public health. In this consideration, we restrict the samples to households with a head under 65 years old and conduct the same estimation methodology as in Table 4 on these subsamples.

Table 9 reports the two-stage estimates of the impact of social networks on households’ access to microcredit and loan amounts, as well as the effect of microcredit on rural public health within the subsample group. From the first-stage estimation, the reciprocal of social fractionalization decreases household microcredit availability and loan amount, while the household social network significantly increases microcredit participation. In the second stage, the regression results show that after the introduction of village-level and household-level instruments of social networks, the microcredit participation of households still positively impacts rural residents’ health. The results of the subsample are consistent with the results of the full sample shown in Table 4.

To further increase our confidence in the estimation, we conducted a battery of robustness checks based on the benchmark ordered Probit model. Although the benchmark results show that microcredit improves the health outcomes of rural residents, many empirical experiences in developing countries have proven that loans or financial stress do not necessarily lead to health improvements. In this section, we introduce three alternative measurements of health: the amount of elder insurance purchased by the household, the coverage of the health insurance and the premium of the health insurance.

$Elder\_ins_{hv}$ denotes the amount of elder insurance purchased by household h, $Ln{\left(coverage\right)}_{hv}$ denotes the health insurance coverage of household h, and $Ln{\left(premium\right)}_{hv}$ denotes the health insurance premium of household h. Table 10 reports the estimates based on these three alternative measurements. Microcredit availability has positive effects on the demand for elder insurance, the improvement in health insurance coverage and the improvement in health insurance premiums. This is consistent with the previous results.

## 5. Discussion

Although this paper further clarifies the relationship between microfinance and health, interesting related topics are expected to be explored in future research. For example, we used poverty reduction and debt amount as mediators in this study. It would be interesting to further our knowledge by studying whether poverty reduction or debt relief could reduce the health problems of the rural public. The link between microcredit availability and social networks is also worthy of future attention. Moreover, since the causal relationship between microcredit and public health may be essentially reversed [8], it would be interesting to study the impact of health on the propensity to acquire (problematic) debt or the two-way spillover effects between health and microcredit. Further, some recent studies have found that having more access to social contacts was associated with better health [63]. Therefore, comprehensive consideration of the microcredit–social network–health relationship is also one research interest for future studies.

It is important for policymakers to understand the relationship between microfinance, debts, health status and health expenditures. Although microfinance has proved to be good for health levels and health expenditure increase, the loans to be paid and the state of poverty post great losses not only to debtors and creditors but also to the whole society, especially the poverty-stricken groups. If the debt burden leads to poor health outcomes, policies to prevent people from over-indebtedness may have a positive external impact and may save on medical and/or financial assistance expenditures. Through the mediation effect tests, our results suggest that debt and poverty may have a substantial negative impact on rural public health and prevent people from obtaining health insurance. Therefore, preventing people from getting excessive debt and alleviating poverty may be an effective way to improve population health.

## 6. Conclusions

Many studies focus on the negative effects of problematic debt on health, while the literature on positive loan–health causality is much sparser. In this study, we focused on the relationship between microcredit availability and health status. We discussed whether obtaining loans from a microfinance program has a positive effect on health. The empirical results from our sample of 11,228 poverty-stricken households suggest that loans from the microfinance program in rural China have improved both the health level and health insurance demand of residents. However, we found that individuals with outstanding loans were more likely to suffer from poor health than those without such problems. Unpaid financial obligations were also associated with lower self-rated health levels and worse health-related behavior. Although microcredit helps improve rural public health, it is still necessary to note that indebtedness might have a serious and lasting negative impact on people’s lives.

The findings reported in this study are important because they show that exogenous income increases from a microfinance program can improve rural public health in developing countries. The results also provide a clear example of the difference in the microcredit effectiveness between high-income groups and low-income groups. Moreover, these findings have immediate policy implications regarding the design of public programs. First, low-interest loans for poor households can increase human capital through health improvement. Relaxing the credit age limit, improving the proportion of long-term credit and expanding credit lines for the poor may be some effective implications for policymakers in China and other developing countries to consider. Second, the identity of microfinance recipients affects its impact. If the program was not naturally favored low-income groups, it would not improve rural public health to the same extent (of course, this does not exclude that loans to high-income groups might affect other types of human capital investment). Therefore, it is very important for the government to innovate accurate poverty identification methods in order to provide microcredit precisely to the poor.

## Figures and Tables

**Table 1:** Descriptive statistics.

Variables	Definition	Mean	S.D.	Min	Max
$Health_{ihv}$	The self-rated health level of the head of household *h* in 2019, which improves from 1 to 4	3.47	0.84	1	4
$Health\_ins_{hv}$	The quantity of health insurance purchased by household *h* in 2019	1.78	2.38	0	9
$Elder\_ins_{hv}$	The quantity of elderly health insurance purchased by household *h* in 2019	0.50	0.75	0	3
$Ln{\left(coverage\right)}_{hv}$	The health insurance coverage of household *h*	12.76	0.28	11.29	13.63
$Ln{\left(premium\right)}_{hv}$	The health insurance premium of household *h*	5.79	0.27	4.09	6.67
$Loan\_dum_{hv}$	Whether or not household h obtained formal credit in 2015; 1 = yes, 0 = no	0.56	0.50	0	1
$Ln{\left(loan\right)}_{hv}$	Logarithm of the formal credit amount of household *h* in 2015, or 0 if without formal credit	6.75	6.10	0	16
$Network_{hv}$	1 = Strong network; 0 = weak network	0.45	0.50	0	1
$Clanship_{v}$	The number of clans in village *v*	2.82	1.75	0	8
$Poverty\_relif_{hv}$	Whether or not household *h* eliminates poverty before 2019	0.39	0.49	0	1
$Ln{\left(debt\right)}_{hv}$	Logarithm of the debt of household h before 2019, or 0 if without debt before 2019	7.92	5.65	0	14.6
$Gender_{ihv}$	Gender of the head of household *h*. Typically, 0 = male; 1 = female	0.09	0.29	0	1
$Age_{ihv}$	Age of the head of household *h*	50.65	11.96	0	1
$Education_{ihv}$	1 = illiterate, 2 = primary graduate, 3 = middle school graduate, 4 = high school graduate, 5 = bachelor, 6 = master	2.47	0.61	1	6
$Capacity_{ihv}$	Capacity level of the head of household *h*. 0 = disabled, 1 = chronic disease, 2 = good labor capacity	0.80	0.42	0	2
$Skill_{ihv}$	The type of skills mastered by the head of household *h*	0.01	0.17	0	4
$Off\_farm_{ihv}$	Whether the head of household *h* is engaged in off-farm work. 0 = no; 1 = yes	0.07	0.26	0	1
$Worktime_{ihv}$	Off-farm working frequency. 0 = no off-farm work, 1 = only in slack seasons, 2 = throughout whole year	0.21	0.49	0	2
$Ln{\left(income\right)}_{hv}$	Logarithm of monthly income of household *h*	8.04	0.12	7.58	10.41
$Poverty_{hv}$	Poverty level of household *h*	2.13	0.94	1	3
$Credit\_score_{hv}$	The credit score level of household *h*. 4 = very high, 3 = high, 2 = low; 1 = very low	2.95	1.22	1	4
$Agri\_ins_{hv}$	The quantity of agricultural insurance purchased by household *h*	0.63	0.95	0	5
$Credit\_ins_{hv}$	The quantity of credit insurance purchased by household *h*	0.05	0.24	0	3
$Interest_{hv}$	Average annualized loan rates of household *h*	3.39	3.18	0	13.2
$Laborperc_{hv}$	The number of labor members of household *h*/the total number of family members of household *h*	0.69	0.32	0	1
$Elderperc_{hv}$	The number of elder members of household *h*/the total number of family members of household *h*	0.23	0.37	0	1

**Table 2:** The effect of microcredit on rural residents’ health.

	Ordered Probit				OLS	
	(1)	(2)	(3)	(4)	(5)	(6)
Variables	$Health_{hv}$	$Health_{hv}$	$Health\_ins_{hv}$	$Health\_ins_{hv}$	$Health_{hv}$	$Health\_ins_{hv}$
$Loan\_dum_{hv}$	0.2579 ***		0.2597 ***			
	(0.0796)		(0.0778)			
$Ln{\left(loan\right)}_{hv}$		0.0369 ***		0.0284 ***	0.0188 ***	0.0325 ***
		(0.0058)		(0.0056)	(0.0029)	(0.0061)
$Gender_{ihv}$	−0.0621	−0.0559	−0.3696 ***	−0.3653 ***	−0.0160	−0.2795 ***
	(0.0408)	(0.0407)	(0.0445)	(0.0445)	(0.0224)	(0.0466)
$Age_{ihv}$	−0.0050 ***	−0.0048 ***	−0.0016	−0.0016	0.0007	−0.0011
	(0.0016)	(0.0016)	(0.0017)	(0.0017)	(0.0008)	(0.0017)
$Education_{ihv}$	−0.0562 **	−0.0583 **	0.0304	0.0285	−0.0020	0.0272
	(0.0238)	(0.0238)	(0.0220)	(0.0220)	(0.0114)	(0.0237)
$Capacity_{ihv}$	1.8466 ***	1.8410 ***	0.1601 ***	0.1541 ***	1.2433 ***	0.1436 ***
	(0.0545)	(0.0545)	(0.0486)	(0.0487)	(0.0225)	(0.0468)
$Skill_{ihv}$	−0.2833 **	−0.2805 **	−0.0207	−0.0192	−0.2293 ***	−0.0376
	(0.1103)	(0.1110)	(0.0785)	(0.0785)	(0.0376)	(0.0784)
$Off\_farm_{ihv}$	0.2631 ***	0.2629 ***	0.0370	0.0362	0.0901 ***	0.0314
	(0.0686)	(0.0687)	(0.0568)	(0.0569)	(0.0280)	(0.0584)
$Worktime_{ihv}$	0.2257 ***	0.2226 ***	−0.0696 **	−0.0722 **	0.0760 ***	−0.0744 **
	(0.0363)	(0.0363)	(0.0291)	(0.0291)	(0.0149)	(0.0311)
$Ln{\left(income\right)}_{hv}$	0.3293 **	0.3223 **	−0.1386	−0.1436	0.1262 **	−0.1666
	(0.1306)	(0.1292)	(0.1021)	(0.1026)	(0.0546)	(0.1137)
$Poverty_{hv}$	−0.1148 ***	−0.1118 ***	−0.1022 ***	−0.0998 ***	−0.0397 ***	−0.1122 ***
	(0.0137)	(0.0138)	(0.0137)	(0.0137)	(0.0070)	(0.0145)
$Credit\_score_{hv}$	0.0360 ***	0.0353 ***	0.0535 ***	0.0531 ***	0.0137 ***	0.0558 ***
	(0.0101)	(0.0101)	(0.0103)	(0.0103)	(0.0052)	(0.0109)
$Agri\_ins_{hv}$	−0.0246 *	−0.0295 **	1.0864 ***	1.0846 ***	−0.0103	1.9944 ***
	(0.0132)	(0.0132)	(0.0185)	(0.0185)	(0.0069)	(0.0143)
$Interest_{hv}$	−0.0113	−0.0365 ***	−0.0150	−0.0261 **	−0.0184 ***	−0.0326 ***
	(0.0122)	(0.0107)	(0.0119)	(0.0105)	(0.0055)	(0.0114)
$Laborperc_{hv}$	−0.4899 ***	−0.4889 ***	−0.2419 ***	−0.2408 ***	−0.1792 ***	−0.1925 ***
	(0.0587)	(0.0587)	(0.0539)	(0.0539)	(0.0271)	(0.0564)
$Elderperc_{hv}$	−0.1122 **	−0.0904 **	0.3779 ***	0.3961 ***	−0.0152	0.4016 ***
	(0.0452)	(0.0454)	(0.0487)	(0.0490)	(0.0250)	(0.0521)
Wald chi2/F	3124.92	3121.09	4289.39	4283.23	-	-
Prob > chi2	0.0000	0.0000	0.0000	0.0000	-	-
Obs.	11,228	11,228	11,228	11,228	11,228	11,228
Adj./Pseudo R^2^	0.2062	0.2076	0.2683	0.2688	0.3716	0.6584

Village-year regressions estimating the effect of microcredit on rural health. The sampling period is 2015 and 2018. In Columns 1, 2 and 5, the dependent variable is the self-rated health level of the household head in 2019. In Columns 3, 4 and 6, the dependent variable is the number of health insurance products purchased by the household in 2019. Robust standard errors are clustered at the village level. The t-statistics are reported in parentheses. The estimation results of two microcredit variables, credit availability and loan amount, are reported to assess the effect of microcredit on rural residents’ health. *, ** and *** indicate significance at the 10%, 5% and 1% levels, respectively.

**Table 3:** The marginal effects of microcredit on rural residents’ health.

Variables		$Loan\_dum_{hv}$	$Ln{\left(loan\right)}_{hv}$
$Health_{hv}$	$Health_{hv}$ = 1	−0.0228 *** (0.0071)	−0.0033 *** (0.0005)
	$Health_{hv}$ = 2	−0.0088 *** (0.0027)	−0.0013 *** (0.0002)
	$Health_{hv}$ = 3	−0.0408 *** (0.0126)	−0.0058 *** (0.0009)
	$Health_{hv}$ = 4	0.0724 *** (0.0223)	0.0103 *** (0.0016)
$Health\_ins_{hv}$	$Health\_ins_{hv}$ = 0	−0.0630 *** (0.0188)	−0.0069 *** (0.0014)
	$Health\_ins_{hv}$ = 1	0.0015 *** (0.0005)	0.0002 *** (0.00004)
	$Health\_ins_{hv}$ = 2	0.0096 *** (0.0029)	0.0010 *** (0.0002)
	$Health\_ins_{hv}$ = 3	0.0147 *** (0.0044)	0.0016 *** (0.0003)
	$Health\_ins_{hv}$ = 4	0.0187 *** (0.0056)	0.0020 *** (0.0004)
	$Health\_ins_{hv}$ = 5	0.0063 *** (0.0019)	0.0007 *** (0.0001)
	$Health\_ins_{hv}$ = 6	0.0046 *** (0.0014)	0.0005 *** (0.0001)
	$Health\_ins_{hv}$ = 7	0.0023 *** (0.0014)	0.0003 *** (0.0001)
	$Health\_ins_{hv}$ = 8	0.0032 *** (0.0011)	0.0003 *** (0.0001)
	$Health\_ins_{hv}$ = 9	0.0020 *** (0.0007)	0.0002 *** (0.0001)

The t-statistics are reported in parentheses. *** indicates significance at the 1% levels.

**Table 4:** Effects of social networks and microcredit on health.

	cmp_oprobit	cmp_oprobit	cmp_oprobit	cmp_oprobit
	(1)	(2)	(3)	(4)
Variables	$Loan\_dum_{hv}$	$Ln{\left(loan\right)}_{hv}$	$Loan\_dum_{hv}$	$Ln{\left(loan\right)}_{hv}$
$Network_{hv}$	0.1971 *** (0.0240)	0.1306 *** (0.0185)	0.1754 *** (0.0214)	0.1355 *** (0.0183)
$Clanship_{v}$	−0.0124 * (0.0071)	−0.0129 *** (0.0040)	−0.0158 *** (0.0059)	−0.0162 *** (0.0039)
	(5)	(6)	(7)	(8)
Variables	$Health_{hv}$	$Health_{hv}$	$Health\_ins_{hv}$	$Health\_ins_{hv}$
$Loan\_dum_{hv}$	0.2380 ***		1.8500 ***	
	(0.0901)		(0.1323)	
$Ln{\left(loan\right)}_{hv}$		0.0078 *		0.0313 ***
		(0.0040)		(0.0077)
Control Variables	Yes	Yes	Yes	Yes
Wald chi2	3229.26	4591.13	11,946.04	11,903.05
Prob > chi2	0.0000	0.0000	0.0000	0.0000
atanhrho	−0.1087 ** (0.0458)	0.1161 *** (0.0401)	−0.7031 *** (0.0478)	0.0062 (0.2376)
Obs.	11,228	11,228	11,228	11,228

Robust standard errors are clustered at the village level. The t-statistics are reported in parentheses. *, ** and *** indicate significance at the 10%, 5% and 1% levels, respectively. The control variables are the same as in Table 2.

**Table 5:** Weak instrumental variable tests.

Endogenous Variables	$Loan\_dum_{hv}$	$Ln{\left(loan\right)}_{hv}$
Cragg–Donald Wald F statistic	32.547 ***	32.778 ***
Kleibergen–Paap rk Wald F statistic	32.598 ***	32.801 ***
Minimum eigenvalue statistic	32.5467	32.7779
Stock–Yogo weak ID test critical values: 10% maximal IV size	19.93	19.93
Stock–Yogo weak ID test critical values: 15% maximal IV size	11.59	11.59

The t-statistics are reported in parentheses. *** indicates significance at the 1% levels.

**Table 6:** Heterogeneous effects of social networks and microcredit on health.

Panel A	High Credit score Group		Low Credit Score Group	
	(1)	(2)	(3)	(4)
Variables	$Health_{hv}$	$Health\_ins_{hv}$	$Health_{hv}$	$Health\_ins_{hv}$
$Loan\_dum_{hv}$	0.3602 ***	1.3400 ***	0.0308	1.5649 ***
	(0.0817)	(0.2552)	(0.1334)	(0.1773)
Control Variables	Yes	Yes	Yes	Yes
Wald chi2	1721.50	3840.73	1507.92	5155.50
atanhrho	−0.1712 ** (0.0742)	−0.5603 *** (0.1288)	0.0430 (0.1055)	−0.7241 *** (0.0956)
Obs.	5916	5916	5312	5312
**Panel B**	**High-income group**		**Low-income group**	
	**(5)**	**(6)**	**(7)**	**(8)**
$Loan\_dum_{hv}$	0.1701 ***	1.3475	0.7578 ***	1.4890 ***
	(0.0628)	(0.2108)	(0.2258)	(0.2128)
Control Variables	Yes	Yes	Yes	Yes
Wald chi2	1994.94	5645.32	1296.53	3247.28
atanhrho	−0.0625 (0.0494)	−0.5438 *** (0.0159)	−0.4706 ** (0.1985)	−0.7355 *** (0.1182)
Obs.	7430	7430	3798	3798
**Panel C**	**Strong network group**		**Weak network group**	
	**(9)**	**(10)**	**(11)**	**(12)**
$Loan\_dum_{hv}$	0.2963 ***	1.6929	−0.5105	−1.5086 ***
	(0.0656)	(0.1723)	(0.3338)	(0.1290)
Control Variables	Yes	Yes	Yes	Yes
Wald chi2	2079.31	5475.88	1304.45	4516.12
atanhrho	−0.1033 ** (0.0486)	−0.7059 *** (0.0845)	0.5623 * (0.3297)	0.9772 *** (0.0591)
Obs.	7116	7116	4112	4112

Robust standard errors are clustered at the village level. The t-statistics are reported in parentheses. ** and *** indicate significance at the 5% and 1% levels, respectively. The control variables are the same as in Table 2.

**Table 7:** The marginal effects of microcredit on rural residents’ health in different groups.

Variables		High Credit Score Group	Low Credit Score Group	High-Income Group	Low-Income Group	Strong Network Group	Weak Network Group
$Health_{hv}$	$Health_{hv}$ = 1	−0.0272 *** (0.0081)	−0.0135 (0.0123)	−0.0135 ** (0.0068)	−0.0452 ** (0.0175)	−0.0307 *** (0.0091)	−0.0106 (0.0111)
	$Health_{hv}$ = 2	−0.0112 *** (0.0034)	−0.0049 (0.0045)	−0.0062 ** (0.0032)	−0.0138 ** (−0.0054)	−0.0107 *** (0.0032)	−0.0049 (0.0051)
	$Health_{hv}$ = 3	−0.0578 *** (0.0169)	−0.0202 (0.0184)	−0.0333 ** (0.0167)	−0.0474 *** (0.0181)	−0.0511 *** (0.0150)	−0.0216 (0.0225)
	$Health_{hv}$ = 4	0.0962 *** (0.0282)	0.0386 (0.0352)	0.0531 ** (0.0267)	0.1064 *** (0.0408)	−0.0926 *** (0.0272)	0.0371 (0.0388)
$Health\_ins_{hv}$	$Health\_ins_{hv}$ = 0	−0.0575 ** (0.0188)	−0.0683 ** (0.0272)	−0.0690 *** (0.0230)	−0.0519 (0.0325)	−0.0263 (0.0303)	−0.0771 *** (0.0240)
	$Health\_ins_{hv}$ = 1	0.0260 ** (0.0005)	0.0022 ** (0.0009)	0.0011 *** (0.0004)	0.0022 (0.0014)	0.0009 (0.0011)	0.0014 *** (0.0005)
	$Health\_ins_{hv}$ = 2	0.0083 ** (0.0038)	0.0109 ** (0.0044)	0.0095 *** (0.0032)	0.0092 (0.0058)	0.0049 (0.0056)	0.0104 *** (0.0033)
	$Health\_ins_{hv}$ = 3	0.0136 ** (0.0062)	0.0154 ** (0.0062)	0.0157 *** (0.0052)	0.0123 (0.0077)	0.0062 (0.0071)	0.0177 *** (0.0055)
	$Health\_ins_{hv}$ = 4	0.0179 ** (0.0081)	0.0192 ** (0.0077)	0.0214 *** (0.0072)	0.0141 (0.0089)	0.0071 (0.0082)	0.0239 *** (0.0075)
	$Health\_ins_{hv}$ = 5	0.0059 ** (0.0027)	0.0067 ** (0.0027)	0.0074 *** (0.0025)	0.0045 (0.0029)	0.0021 (0.0024)	0.0085 *** (0.0027)
	$Health\_ins_{hv}$ = 6	0.0038 ** (0.0018)	0.0059 ** (0.0024)	0.0054 *** (0.0019)	0.0034 (0.0022)	0.0019 (0.0022)	0.0057 *** (0.0018)
	$Health\_ins_{hv}$ = 7	0.0024 ** (0.0011)	0.0020 ** (0.0010)	0.0026 *** (0.0009)	0.0020 (0.0014)	0.0009 (0.0010)	0.0030 *** (0.0011)
	$Health\_ins_{hv}$ = 8	0.0027 ** (0.0013)	0.0039 *** (0.0018)	0.0036 *** (0.0013)	0.0025 (0.0017)	0.0012 (0.0014)	0.0042 *** (0.0015)
	$Health\_ins_{hv}$ = 9	0.0019 ** (0.0010)	0.0021 * (0.0011)	0.0023 ** (0.0009)	0.0017 (0.0013)	0.0011 (0.0013)	0.0022 ** (0.0009)

The t-statistics are reported in parentheses. *, ** and *** indicate significance at the 10%, 5% and 1% levels, respectively.

**Table 8:** Mediation effects of debt and poverty reduction in the microcredit–health relationship.

Dependent Variables								
$Health_{hv}$					$Health\_ins_{hv}$			
Mediator	$Ln{\left(debt\right)}_{hv}$		$Poverty\_relif_{hv}$		$Ln{\left(debt\right)}_{hv}$		$Poverty\_relif_{hv}$	
Model	Ologit	Oprobit	Ologit	Oprobit	Ologit	Oprobit	Ologit	Oprobit
Reduced	0.0621 *** (0.0100)	0.0362 *** (0.0058)	0.0681 *** (0.0101)	0.0391 *** (0.0058)	0.0928 *** (0.0084)	0.0551 *** (0.0049)	0.0462 *** (0.0101)	0.0305 *** (0.0057)
Full	0.0728 *** (0.0107)	0.0415 *** (0.0062)	0.0649 *** (0.0101)	0.0375 *** (0.0058)	0.0566 *** (0.0090)	0.0339 *** (0.0053)	0.0440 *** (0.0101)	0.0291 *** (0.0057)
Diff	−0.0106 *** (0.0040)	−0.0053 ** (0.0023)	0.0032 *** (0.0009)	0.0016 *** (0.0004)	0.0362 *** (0.0039)	0.0211 *** (0.0023)	0.0022 *** (0.0007)	0.0015 *** (0.0004)
Conf_ratio	0.8539	0.8720	1.0089	1.0082	1.6408	1.6233	1.0062	1.0059
Conf_Pct	−17.11	−14.68	0.88	0.81	39.05	38.40	0.62	0.59

The t-statistics are reported in parentheses. ** and *** indicate significance at the 5% and 1% levels, respectively. The control variables are the same as in Table 2.

**Table 9:** Effects of social networks and microcredit on health (subsample regression results).

	cmp_oprobit	cmp_oprobit	cmp_oprobit	cmp_oprobit
Variables	$Loan\_dum_{hv}$	$Ln{\left(loan\right)}_{hv}$	$Loan\_dum_{hv}$	$Ln{\left(loan\right)}_{hv}$
$Network_{hv}$	0.1445 *** (0.0261)	0.0813 *** (0.0208)	0.1481 *** (0.0248)	0.0824 *** (0.0193)
$Clanship_{v}$	−0.0019 (0.0078)	−0.0039 (0.0050)	−0.0081 (0.0073)	−0.0090 ** (0.0043)
	(5)	(6)	(7)	(8)
Variables	$Health_{hv}$	$Health_{hv}$	$Health\_ins_{hv}$	$Health\_ins_{hv}$
$Loan\_dum_{hv}$	0.1573 **		1.2565 ***	
	(0.0771)		(0.2407)	
$Ln{\left(loan\right)}_{hv}$		0.0098 **		0.0091 **
		(0.0038)		(0.0041)
Wald chi2	4447.22	4038.27	10,229.97	4342.06
Prob > chi2	0.0000	0.0000	0.0000	0.0000
atanhrho	−0.0129 (0.0660)	0.0729 ** (0.0286)	−0.4299 *** (0.1011)	0.0934 *** (0.0320)
Obs.	9764	9764	9764	9764

Robust standard errors are clustered at the village level. The t-statistics are reported in parentheses. ** and *** indicate significance at the 5% and 1% levels, respectively. The control variables are the same as in Table 2.

**Table 10:** The effects of microcredit on other types of health insurance.

	OLS	OLS	OLS	Oprobit	Tobit	Tobit
	(1)	(2)	(3)	(4)	(5)	(6)
Variables	$Elder\_ins_{hv}$	$Ln{\left(coverage\right)}_{hv}$	$Ln{\left(premium\right)}_{hv}$	$Elder\_ins_{hv}$	$Ln{\left(coverage\right)}_{hv}$	$Ln{\left(premium\right)}_{hv}$
$Loan\_dum_{hv}$	0.0443 ***	0.0699 ***	0.0727 ***	0.3734 ***	0.0699 ***	0.0727 ***
	(0.0146)	(0.0143)	(0.0135)	(0.1027)	(0.0147)	(0.0138)
Control variables	Yes	Yes	Yes	Yes	Yes	Yes
Observations	11,228	11,228	11,228	11,228	11,228	11,228
Adj./Pseudo R2	0.8592	0.2287	0.2854	0.6949	0.9491	1.4922
	(7)	(8)	(9)	(10)	(11)	(12)
	2SLS	2SLS	2SLS	Cmp-oprobit	IV-Tobit	IV-Tobit
$Loan\_dum_{hv}$	1.4347 ***	1.3175 ***	1.1172 ***	0.0870 ***	1.3172 ***	1.1176 ***
	(0.4795)	(0.4385)	(0.3945)	(0.0176)	(0.4411)	(0.3974)
Control variables	Yes	Yes	Yes	Yes	Yes	Yes
Wald chi2	-	-	-	49,236.88	2019.49	2954.79
Observations	11,228	11,228	11,228	11,228	11,228	11,228

Robust standard errors are clustered at the village level. The t-statistics are reported in parentheses. *** indicates significance at the 1% level. The control variables are the same as in Table 2.

## Data Availability

The data were collected through micro survey from Yanchi County government statistical data of the officially registered poverty-stricken households in the year of 2015, 2018 and 2019.
